# Supplementary material for: Exploring the potential impact of medical errors research on population health
Source: PLoS One. 2026 Mar 12;21(3):e0340153. doi: 10.1371/journal.pone.0340153 (PMC12981467; doi:10.1371/journal.pone.0340153)
Supplement: S1 File — (DOCX) [file pone.0340153.s001.docx]

**Full search strategies**

Reproduced July 11, 2024

**Scopus**

SUBJAREA(HEAL) OR SUBJAREA(DENT) OR SUBJAREA(NURS) OR SUBJAREA(MEDI) OR SUBJAREA(BIOC) OR SUBJAREA(IMMU) OR SUBJAREA(NEUR) OR SUBJAREA(PHAR) AND TITLE("Medical Errors") OR TITLE("Diagnostic Errors") OR TITLE("Medication Errors"): 7169 results

**PubMed**

("Medical Errors"[Title] OR "Diagnostic Errors"[Title] OR "Medication Errors"[Title]) = 4411 results

**Web of Science Core Collection**

(TI=("Medical Errors" OR "Diagnostic Errors" OR "Medication Errors")) OR (AB=("Medical Errors" OR "Diagnostic Errors" OR "Medication Errors")) and Health Care Sciences Services or Pharmacology Pharmacy or Medicine General Internal or Nursing or Health Policy Services or Public Environmental Occupational Health or Pediatrics or Medical Informatics or Surgery or Radiology Nuclear Medicine Medical Imaging or Emergency Medicine or Anesthesiology or Oncology or Clinical Neurology or Toxicology or Critical Care Medicine or Medicine Research Experimental or Pathology or Obstetrics Gynecology or Psychiatry or Geriatrics Gerontology or Cardiac Cardiovascular Systems or Primary Health Care or Medical Laboratory Technology or Infectious Diseases or Neurosciences or Orthopedics or Gerontology or Dermatology or Urology Nephrology or Respiratory System or Hematology or Dentistry Oral Surgery Medicine or Endocrinology Metabolism or Gastroenterology Hepatology or Peripheral Vascular Disease or Social Sciences Biomedical or Medicine Legal or Ophthalmology or Psychology or Immunology or Otorhinolaryngology or Rheumatology or Medical Ethics or Tropical Medicine or Genetics Heredity or Neuroimaging or Virology or Parasitology or Anatomy Morphology or Physiology (Web of Science Categories) = 6247 results

**SciELO Citation Index**

(TI=("Medical Errors" OR "Diagnostic Errors" OR "Medication Errors")) OR (AB=("Medical Errors" OR "Diagnostic Errors" OR "Medication Errors")) and Nursing or Pharmacology Pharmacy or Medicine General Internal or Health Care Sciences Services or Public Environmental Occupational Health or Pediatrics or Health Policy Services or Anesthesiology or Radiology Nuclear Medicine Medical Imaging or Surgery or Medicine Research Experimental or Infectious Diseases or Medicine Legal or Medical Ethics or Cardiac Cardiovascular Systems or Medical Laboratory Technology or Neurosciences or Anatomy Morphology or Ophthalmology or Urology Nephrology or Critical Care Medicine or Dentistry Oral Surgery Medicine or Gastroenterology Hepatology or Nutrition Dietetics or Pathology or Psychiatry or Tropical Medicine or Oncology or Rheumatology or Allergy or Clinical Neurology or Geriatrics Gerontology or Immunology or Medical Informatics or Obstetrics Gynecology or Orthopedics or Otorhinolaryngology or Parasitology or Peripheral Vascular Disease (SciELO Categories) = 345 results

**KCI-Korean Journal Database**

(TI=("Medical Errors" OR "Diagnostic Errors" OR "Medication Errors")) OR (AB=("Medical Errors" OR "Diagnostic Errors" OR "Medication Errors")) and Nursing Science or Preventive Medicine Occupational And Environmental Medicine or Pharmacy or General Medicine or Medicine And Pharmacy or Emergency Medicine or Radiology or Pathology or Other Medicine And Pharmacy or Anesthesiology or Family Medicine or Dentistry or Neurology or Obstetrics And Gynecology or Orthopedic Surgery or Otorhinolaryngology or Urology (KCI-KJD Categories) = 123 results
