# Supplementary material for: Exploring the potential impact of medical errors research on population health
Source: PLoS One. 2026 Mar 12;21(3):e0340153. doi: 10.1371/journal.pone.0340153 (PMC12981467; doi:10.1371/journal.pone.0340153)
Supplement: S5 File — (DOCX) [file pone.0340153.s005.docx]

**
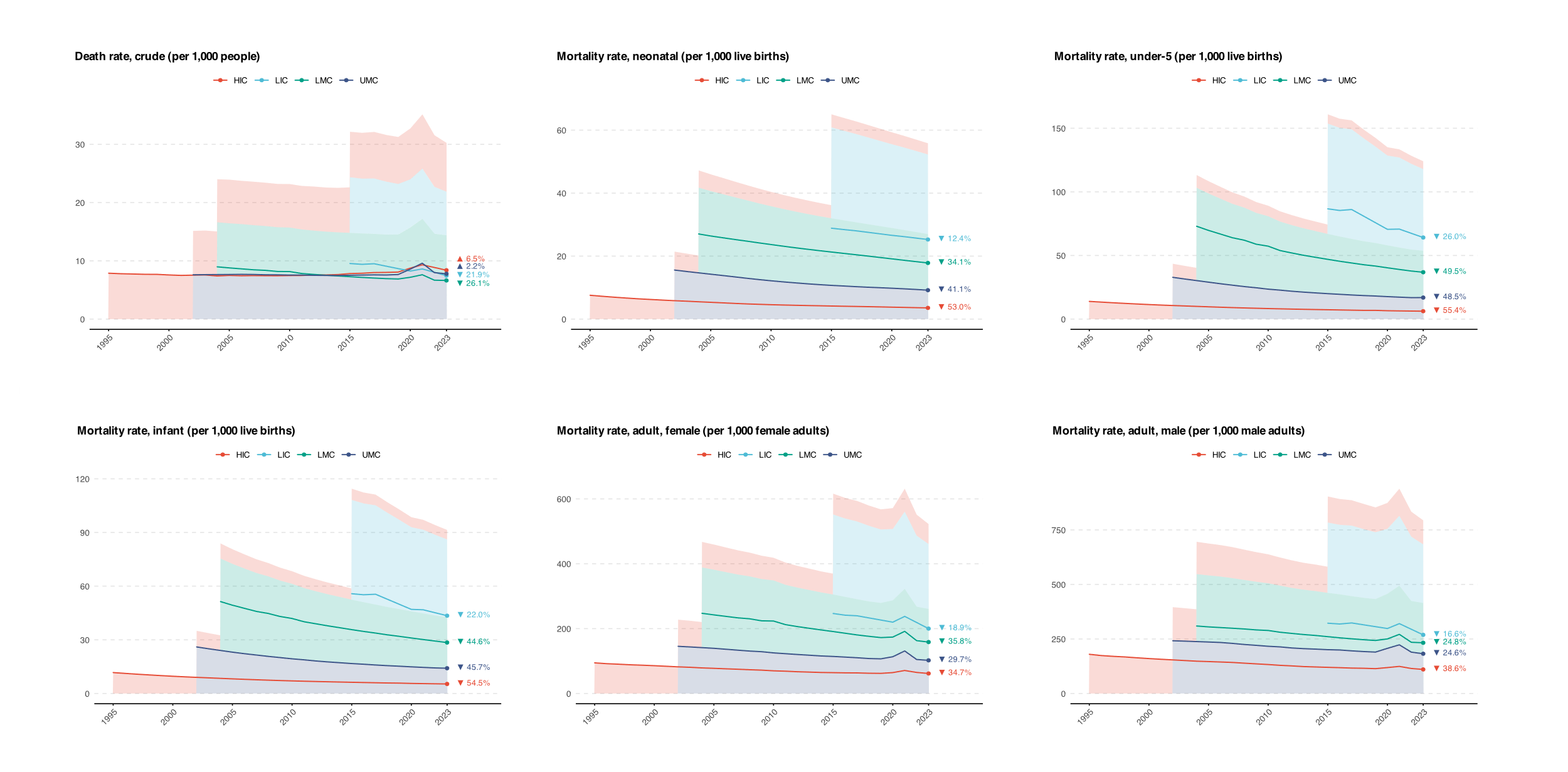
Supplementary Material 5**

**Figure S1. Temporal Trends in Mortality Indicators Across Country Income Classifications.** Time series showing trends in six mortality indicators across four World Bank income classifications (HIC: high-income countries, UMC: upper-middle-income countries, LMC: lower-middle-income countries, LIC: low-income countries). Lines represent mean values per income group with semi-transparent areas indicating data range. Percentage values and directional indicators (▲ increase, ▼ decrease) at line endpoints quantify relative change between earliest and latest available data points.

**
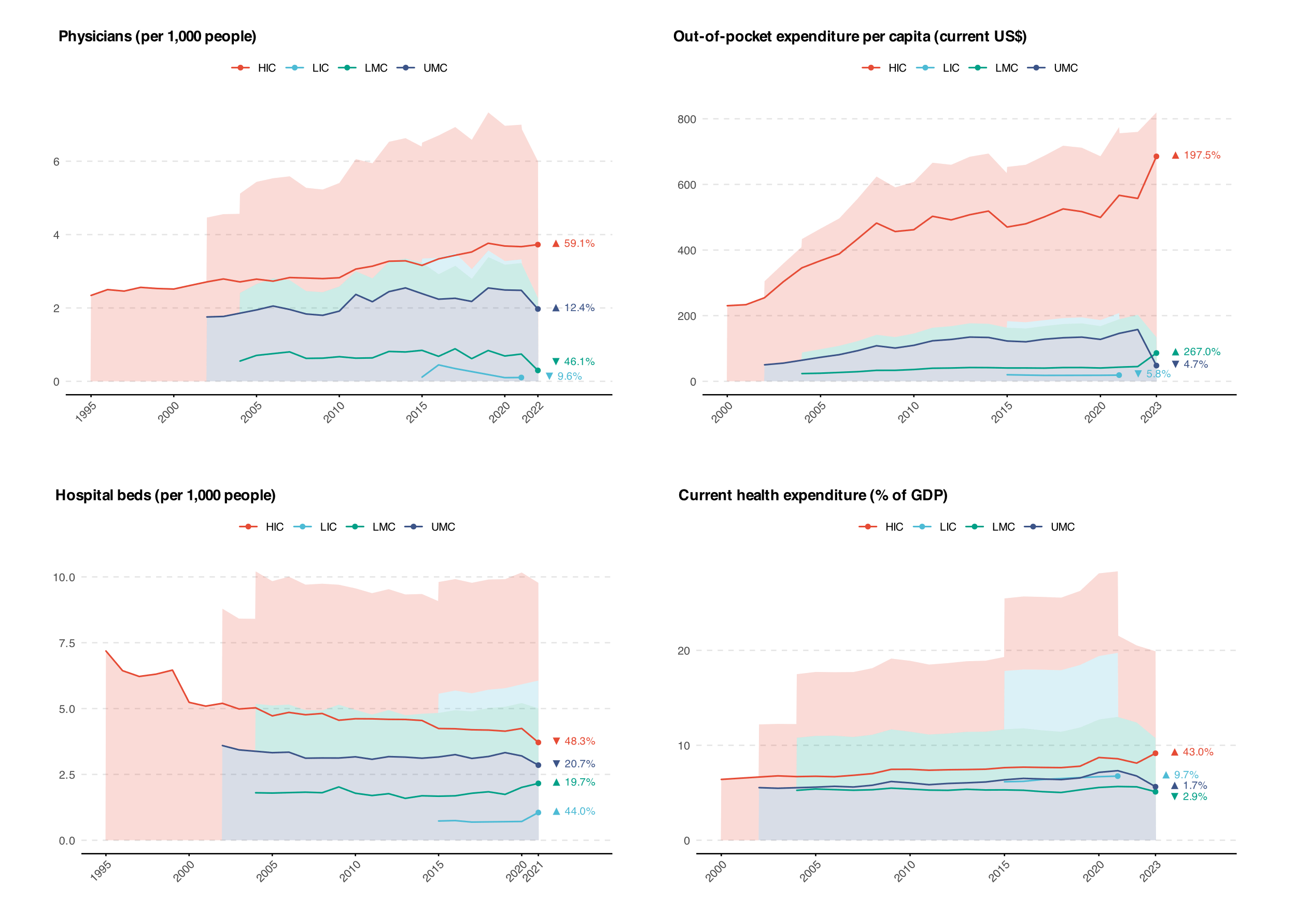
**

**Figure S2. Temporal Trends in Health System Indicators Across Country Income Classifications.** Time series showing trends in four health system indicators across World Bank income classifications (HIC: high-income countries, UMC: upper-middle-income countries, LMC: lower-middle-income countries, LIC: low-income countries). Lines represent mean values with semi-transparent areas indicating data range. Percentage values and directional indicators (▲ increase, ▼ decrease) quantify relative change between earliest and latest available data points.


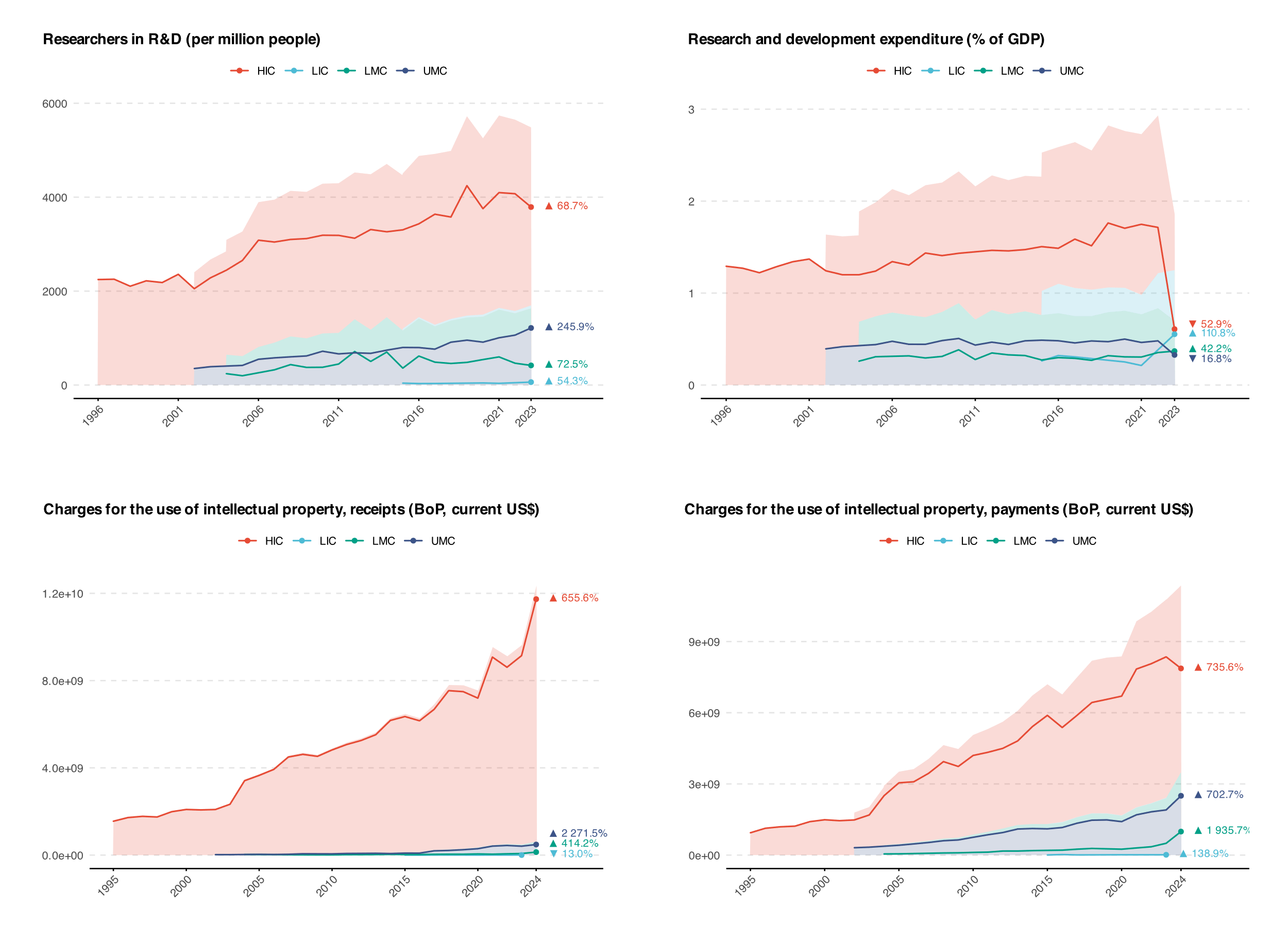


**Figure S3. Temporal Trends in Research & Development and Innovation Indicators Across Country.** Time series showing trends in four R&D and innovation indicators across World Bank income classifications (HIC: high-income countries, UMC: upper-middle-income countries, LMC: lower-middle-income countries, LIC: low-income countries). Lines represent mean values with semi-transparent areas indicating data range. Percentage values and directional indicators (▲ increase, ▼ decrease) quantify relative change between earliest and latest data points.


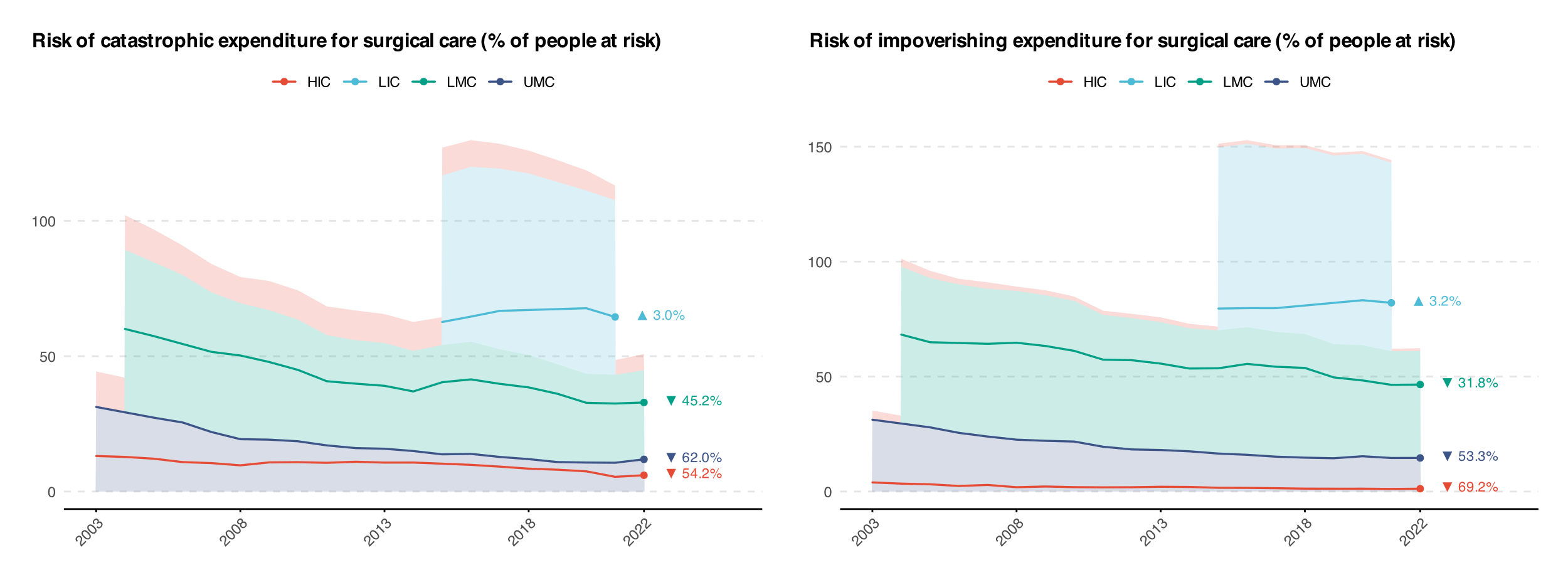


**Figure S4. Temporal Trends in Financial Risk Indicators for Surgical Care Across Country Income Classifications.** Time series showing trends in financial risk indicators for surgical care across World Bank income classifications (HIC, UMC, LMC, LIC). Lines represent mean values with semi-transparent areas indicating data range. Percentage values and directional indicators (▲ increase, ▼ decrease) quantify relative change between earliest and latest data points.
