# Supplementary material for: Exploring the potential impact of medical errors research on population health
Source: PLoS One. 2026 Mar 12;21(3):e0340153. doi: 10.1371/journal.pone.0340153 (PMC12981467; doi:10.1371/journal.pone.0340153)
Supplement: Attachment — Submitted filename: Answer to reviewers.docx [file pone.0340153.s006.docx]

**Answer to reviewers**

**Manuscript ID: PONE-D-25-28355**

**Association of Medical Errors-Related Publications with Global Health and Research and Development Indicators: A Scientometrics Study Focus on Countries by Income**

**New manuscript ID: PONE-D-25-42260**

**New title: Exploring the Potential Impact of Medical Errors Research on Population Health, Health System, and Research and Development Indicators**

*We are grateful with the Editor and Reviewers for the expert reading of the manuscript. We have found the comments appropriate and helpful. They have permitted us to improve the manuscript. Appropriate changes were made and highlighted in the revised manuscript according to suggestions. Following are the responses to the comments.*

**Reviewer #1**

**The overall topic covered in this study is important and interesting. However, the specific approach adopted here raises many doubts.**

**My two main questions are the following ones:**

**- Why is there a causal relationship between the production of scientific research and health results? From my point of view, the problem is much more complex. The country-based approach does not makes full sense, in my opinion. Additionally, the casual relationship seems to be with GDP (or GDP per capita).**

***R/*** We thank the reviewer for this insightful question. We acknowledge that our earlier manuscript version may have implied causal relationships where none were definitively established. In response, we have completely reformulated our conceptual framework to clarify that we are examining theoretical coherence between research production and its potential translation into outcomes, rather than direct causation.

We selected indicators most conceptually related to medical errors to make these inferences, recognizing that no directly-related indicators exist for this topic. We understand that a complex chain of processes mediates between research publication and measurable population outcomes. In this revised version, we have explicitly clarified the proxy nature of our analytical framework, which we've also strengthened to reduce false discovery rates.

Our aim is to contribute to broader discussions on health research relevance, accountability, and equity in global contexts. While one might expect increased research to correlate with improved outcomes, instances where our analyses don't reflect this relationship, or show the reverse, are precisely the points worthy of critical discussion.

**- Accepting the relevance of the question and the validity of the conclusions, why the implication for policy action is not to promote a better diffusion of research outputs - Open access strategy in the case of some countries?**

***R/*** We sincerely thank the reviewer for this valuable suggestion. Your comment has highlighted an important policy implication that complements our findings, which we have now incorporated into the revised manuscript.

In our updated discussion, we have expanded on how the observed inequities in research influence across income levels could be addressed through improved dissemination mechanisms. Specifically, we have strengthened our analysis of the "disconnection" between research production and implementation in lower-income settings, noting that open access publication models represent a critical policy intervention to bridge this gap.

Our findings regarding the uneven distribution of benefits from medical error research, particularly the observation that middle-income countries show promising associations between publication activity and outcomes, provide compelling evidence for open access strategies. We now emphasize that knowledge translation barriers could be significantly reduced through policies promoting unrestricted access to research, especially in settings where institutional subscriptions to scientific journals are limited by financial constraints. This addition aligns with our call for "building capacity for research production, dissemination, and use in LMICs and LICs" and addresses the "equity gap" we identified.

**Moreover, the paper could be improved with the existence of a (short) literature review.**

***R/*** We appreciate the suggestion. Considering that the manuscript was restructured and certain concepts were further developed, the literature review was expanded, allowing, in our view, for a better understanding of the concepts and their comparison with arguments related to global health and health systems.

**Reviewer #2**

**Thank you for the opportunity to review this article. The authors address an important and timely question, but there are several methodological concerns that warrant attention before the findings can be meaningfully interpreted. I have included some specific comments in the PDF and have these general thoughts:**

**• It appears there may be some misunderstandings in the application of certain scientometrics measures. For example, the manuscript refers to the h-index of articles, but this is a metric used to evaluate authors, not publications.**

***R/*** We thank the reviewer for this insightful comment. We acknowledge the misunderstanding regarding the h-index and have addressed this in our revised manuscript. To clarify, the h-index we referred to was not for individual articles (as this indeed does not exist), but for the journals in which the articles were published, according to the Scimago Journal & Country Rank and Journal Citation Reports for the respective year of publication.

In this new version, we have made this distinction explicit throughout the manuscript. Additionally, we have simplified our approach to modeling potential relationships with population-level indicators by using only the number of publications as the predictive/outcome variable. The citation counts and journal h-indices are now used exclusively in the descriptive bibliometric analyses, rather than in the inferential models.

**The description of selecting “the highest-ranking metric for the journal in which each document was published” on page 8 is also unclear.**

***R/*** We thank the reviewer for highlighting this unclear description. We have revised this section to provide greater clarity about our methodology. In the updated version, we explicitly state that we collected the journal's quartile ranking and h-index for each publication, according to the publication year, from both Scimago Journal & Country Rank and Journal Citation Reports. We have clarified that when metrics were available from both sources, we selected the highest-ranking metric.

**Overall the discussion of scientometric and bibliometric measures makes it difficult to assess how these metrics were applied and raises concerns about whether these indicators have been used rigorously and appropriately. Additionally, the discussion section appears to frame higher bibliometric scores as inherently desirable outcomes. While these measures can reflect academic attention (e.g., citations), they do not necessarily correspond to improvements in clinical practice or reductions in medical errors, which is the focus of this article.**

***R/*** We thank the reviewer for this important comment. In this new version of the manuscript, we have substantially revised our approach and discussion in several key ways:

First, we simplified our regression models to use only the number of publications as the variable of interest, limiting citations and h-index data to descriptive bibliometric analyses only.

Second, we have completely reformulated our conceptual framework to clarify that we are examining theoretical coherence between research production and its potential translation into outcomes, rather than direct causation. We understand that bibliometric measures are not inherently desirable outcomes in themselves, but rather proxies within a complex chain that may connect knowledge production to clinical improvements.

In our revised discussion, we explicitly acknowledge the proxy nature of our analytical framework, recognizing that no directly-related indicators exist for medical errors outcomes. We selected indicators most conceptually related to this topic to make inferences while strengthening our methods to reduce false discovery rates.

Our aim is to contribute to broader discussions on health research relevance, accountability, and equity in global contexts. While one might expect increased research to correlate with improved outcomes, instances where our analyses don't reflect this relationship, or show the reverse, are precisely the points worthy of critical discussion. The revised discussion now focuses on these relationships rather than framing bibliometric success as an end goal.

This approach aligns with the reviewer's concern that academic attention metrics do not necessarily correspond to improvements in clinical practice or reductions in medical errors, which is indeed the primary focus of our article.

**• The authors include a large number of healthcare quality indicators and global health metrics and develop statistical models examining their relationship to publication metrics by country income group. However, this approach seems to prioritize identifying statistically significant associations without a clear theoretical basis. With a large enough number of models, some statistically significant results are likely to appear by chance. In this particular study, many of the associations identified are likely confounded by country income level. For example, the statement “Each additional publication corresponded to reductions of 0.424 male deaths and 0.241 female deaths per 1,000 adults” suggests a causal relationship between publication counts and decreased mortality, which is unlikely. It seems more plausible that a third factor, country income level, influences both research output and health outcomes. While strengthening research capacity in lower-middle- and low-income countries is absolutely an important and worthwhile goal, the statistical models presented in this manuscript lack a clear theoretical foundation, and the relationships identified are not convincingly explained or supported by the underlying data.**

***R/*** We thank the reviewer for these insightful comments. We agree that our initial approach had significant limitations regarding multiple testing and potential confounding by income level. In response, we have completely reformulated our analytical framework with several methodological enhancements:

First, to address concerns about multiple testing and chance findings, we implemented the Benjamini-Hochberg method to control the false discovery rate, with an adjusted p-value threshold of 0.05 for statistical significance. This substantially reduces the likelihood of identifying spurious associations.

Second, we developed a more rigorous approach to model selection. Rather than applying a uniform analytical method, we employed an adaptive modeling strategy based on data characteristics - using Poisson regression for equidispersed count data, negative binomial for overdispersed counts, beta regression for proportional data, and so forth. Each model was selected based on the specific distribution of the variables being analyzed.

Third, to directly address confounding by country income level, we implemented hierarchical mixed-effects models with income group as a random effect. This approach explicitly accounts for clustering within economic classifications while still allowing us to examine relationships between research output and health indicators. For these models, we required a minimum of 20 observations to ensure adequate statistical power.

Fourth, all models included publication year as a covariate to account for temporal trends, and we conducted systematic model diagnostics including convergence assessment and residual analysis.

Regarding theoretical foundation, we have restructured our conceptual framework to highlight the proxy nature of our analyses, explicitly acknowledging that we do not measure causal relationships. Instead, we are testing the hypothesis of "scientific coherence" - the theoretical expectation that research production should translate into improved outcomes. Our revised approach examines whether this expected relationship holds true across different contexts, recognizing that statistical associations do not imply causation. We critically discuss instances where the data don't support this assumption, as well as where they do. Rather than focusing solely on statistically significant results, we now consider the broader patterns, including non-significant and contrary findings, as equally important for understanding the complex relationship between knowledge production and health outcomes.

**• I appreciate the authors’ inclusion of their R code, but I was unable to locate the underlying dataset, including the list of publications analyzed. Providing access to this dataset (at minimum, the publication list) is important to ensuring the transparency and reproducibility of the analysis. I would recommend including it as a supplementary file, or perhaps including it in the Zenodo repository with the R script.**

***R/*** We thank the reviewer for this valuable suggestion. We have not included the full list of articles as a supplementary file, but have instead enhanced the transparency and reproducibility of our methodology. Specifically, we have comprehensively improved our methods section by clarifying the search strategy and adding it as supplementary material, detailing inclusion and exclusion criteria, and including a PRISMA flow diagram that documents the entire article selection process. The complete list of analyzed articles is in our possession and can be provided upon reasonable request. These enhancements provide clear guidance for any researcher who wishes to replicate our search and selection process, addressing the core concern about reproducibility while maintaining focus on our methodological approach.

**Reviewer #3**

**The study attempts to address an interesting problem related to understanding the relationship between metrics of medical error scholarship and regional health outcomes and the role of regional differences in income level. Unfortunately, the specific research questions are insufficiently specified to have set up clear research methods and appropriate analytic approaches. A more fulsome consideration of the background informing this study to clarify why national research output on medical errors should be connected to local health outcomes would have helped narrow the focus of what variables to focus on as the basis for the research questions.**

***R/*** We thank the reviewer's suggestion. We agree and hence we have completely reframed the conceptual framework of our study to address these important concerns. In this revised version, we have:

1. Developed a more explicit theoretical foundation based on "scientific coherence" - the premise that knowledge production should theoretically translate to practice improvements and eventually to measurable outcomes. This provides a clearer rationale for examining relationships between research output and health indicators.
2. More precisely specified our research questions to focus on whether patterns of medical error research correspond with health system performance indicators across different economic contexts, rather than assuming direct causal links.
3. Clarified the proxy nature of our analyses throughout the manuscript. We now explicitly acknowledge that we are not measuring causal relationships, but rather examining potential associations that may reflect complex knowledge translation pathways.
4. Implemented hierarchical mixed-effects models that explicitly account for income level as a structuring factor, enabling us to better distinguish patterns within economic contexts from those between them.

**The role of regional categories of income level in the relationship is not fully explored. It is unclear if its role is as the primary IV that impacts research output which in turn relates to health outcomes. Or if it is a moderating variable that affects the strength of the relationship between research metrics and health outcomes. In addition, the background does not discuss how relevant national/regional contextual factors may act as covariates to consider exploring.**

***R/*** We thank the reviewer for this important conceptual clarification. We recognize that our manuscript did not sufficiently articulate the theoretical role of income level in our analytical framework. In the revised version, we have explicitly addressed this gap by:

1. Clarifying that our two-stage analytical approach directly examines the dual role of income level: first as a stratifying variable (to identify distinct patterns within economic contexts) and then as a moderating variable in hierarchical models (to test whether relationships persist across economic contexts). This approach allows us to systematically assess whether income level primarily influences research production, moderates the relationship between research and outcomes, or both.
2. Expanding our introduction to explicitly address how income level moderates knowledge translation pathways, stating: " This question is particularly pressing in low- and middle-income countries (LMICs), where limited research output may reflect broader systemic barriers, and where the consequences of inaction can be most severe. On the other hand, in high-income countries (HICs) with greater resources, the assumption that research leads to impact remains largely untested across macro-level indicators."
3. Adding discussion of how relevant contextual factors beyond income classification (such as healthcare system structure, research infrastructure, and policy implementation capacity) may act as important covariates influencing the relationship between research production and outcomes. These factors are now addressed in our limitations section where we acknowledge that "associations observed may be influenced by unmeasured confounders such as health policy reforms or institutional practices not captured in national indicators."

Our revised approach makes explicit that we consider income level primarily as a moderating factor that may alter the strength and nature of relationships between research output and health outcomes, rather than assuming a simple causal pathway from income to research to outcomes.

**Describing the role of the income variable and how other contextual variables might impact the basic relationship and how these ideas have already been studied needs to be added to the background which may help set up more well specified research questions. For example, a more explicit question that has emerged from my reading of this work is about the association between these research metrics and relative investment into health programs as measured by something like proportion of the GDP dedicated to healthcare spending or healthcare spending of specific type. This relationship speaks more directly to the problem being laid out than exploring the relationship between research metrics and health outcomes which ignores so many factors that could be at play beyond the presence or absence of medical error research.**

***R/*** We thank the reviewer for these insightful comments. We recognize the complexity in connecting research publications to health outcomes, such as mortality rates, as this relationship involves numerous intermediate steps and processes. However, this challenge extends equally to examining connections between publications and healthcare spending indicators such as the proportion of GDP dedicated to healthcare, as you mentioned.

It is due to this high variability and third variables involved in this translation that we have remarked in our conceptual framework that we do not state causal associations. Instead, we estimate potential impacts or interactions between these variables, based on the idea of "scientific coherence": the theoretical expectation that more research translates into better outcomes. However, whether this expectation is fulfilled or not is the core of our critical discussion.

Hence, since indicators such as GDP dedicated to healthcare and health outcomes have relatively the same level of uncertainty and bias, we opted for including all these domains that we thought could be expected to influence or be influenced by research output on medical errors. This approach encompasses a broad landscape of empirical evidence of the population-level potential consequences of medical error research, since this theoretical framework has not been described in the literature before specifically for this topic, to our knowledge.

**There are multiple problems with the methods of the study. Most critically with the analysis. Running 264 individual simple linear regressions on outcomes of different types (proportions, counts, and continuous variables) without accounting for the hierarchical nature of the data results in numerous threats to statistical conclusion validity. This approach assumes normally distributed residuals, unbounded and homoscedastic (equal variance). Count outcomes should use Poisson or Negative Binomial regression. Proportions should be modeled using logit or beta regression. Using linear regression with these types of data violates model assumptions, producing biased coefficients, invalid standard errors, and incorrect p-values/confidence intervals. There is also a problem of overfitting for subgroups with small sample sizes like is present with the low income category that had only 11 studies. There is also a type 1 error problem with the multiple (264) comparisons that has not been accounted for. All of these issues lead to inappropriate inferences. Further, the meta-analytic approach to the regression results is also problematic because the coefficients used for its basis are problematic. Hierarchical multiple regression analyses for each outcome that includes relevant country level covariates would have produced simple unified results for each outcome that were statistically defensible and a valid basis for interpretation. This would have facilitated presenting the model outputs in tables organized into related clusters so that a discussion of the findings would have been more intuitive and able to speak to differences across types of outcomes. In addition, the income level variable could have been included as a simple main effect in all the models with a reduced the number of categories so that the cell sizes were larger for the bottom category.**

***R/*** We thank the reviewer for these critical methodological insights. We have completely restructured our statistical approach to address each concern raised:

1. **Inappropriate regression types:** We now employ an adaptive modeling approach that matches regression type to data characteristics:
   - Poisson regression for equidispersed count data
   - Negative binomial regression for overdispersed count data
   - Beta regression for continuous proportions
   - Linear regression only for normally distributed continuous variables
2. **Hierarchical data structure:** We've implemented hierarchical mixed-effects modeling with income group as a random effect, which properly accounts for clustering within economic classifications while estimating both fixed and random effects.
3. **Overfitting in small samples:** We've established minimum thresholds (5 observations for stratified analyses, 20 for hierarchical models) to ensure adequate statistical power. The hierarchical approach also mitigates small sample issues by borrowing strength across income groups.
4. **Multiple comparison problem:** We now apply the Benjamini-Hochberg method to control false discovery rate across all analyses, with significance determined at an adjusted p-value threshold of 0.05.
5. **Meta-analytic concerns:** We've completely replaced this approach with hierarchical mixed-effects modeling, which provides a more rigorous framework for synthesizing findings across groups.
6. **Unified modeling framework:** As suggested, we now implement hierarchical models for each outcome that include publication year as a covariate and income level as a random effect, producing more statistically defensible results that can be interpreted across outcome types.

The revised statistical approach provides a more rigorous, appropriate, and transparent analytical framework that addresses all the methodological concerns raised. The complete analysis with code and detailed results is available in our public repository for review.

**The description of the identification, screening and selection of articles for inclusion is also too vague to be replicated. More objective definitions of the criteria and a PRISMA flow diagram showing how the database of studies was created should be added.**

***R/*** We thank the reviewer for this important recommendation. We have comprehensively enhanced our methodology section by adding clear inclusion and exclusion criteria definitions, detailed screening process description, a complete PRISMA flow diagram (Figure 1), and the full search strategy in Supplementary Material 1. These enhancements ensure full transparency and replicability of our study selection process.

**Finally, the measures (metrics) employed in the study need to be mapped to the main variables of the study (i.e. medical error research and population health) and these main variables should be defined nominally (conceptually) so we understand the dimensions involved. This could set up the identification of health outcome subcategories to organize the metrics and subsequent results to provide a meaningful basis to discuss differences. I suspect that results differ depending on the type of health outcome being considered. All metrics need operational definitions so that readers know how they were measured including the timing of when they were collected.**

***R/*** We thank the reviewer for these insightful comments. We have comprehensively addressed each point as follows:

First, we have defined our main variables (medical error research and population health) conceptually in the methods section, clarifying the dimensions involved in each construct to provide a stronger theoretical foundation.

Second, we have enhanced our conceptual framework to better discuss the results, properly connecting each indicator domain with the topic of medical errors research.

Third, we have now categorized the indicators into four distinct domains (financial surgical risk, health system, mortality and R&D/innovation indicators) and structured our results reporting based on these categories to facilitate meaningful interpretation.

Fourth, we have included raw data for all indicators along with bibliometric measures in Supplementary File S2. This data is aggregated by year and income group to ensure complete transparency. Additionally, we clarified the methodology used for retrieving these population-level indicators in both the methods section and the R script available on Zenodo.
